# Observation of optomechanical buckling transitions

**DOI:** 10.1038/ncomms14481

**Published:** 2017-03-01

**Authors:** H. Xu, U. Kemiktarak, J. Fan, S. Ragole, J. Lawall, J. M. Taylor

**Affiliations:** 1 Joint Quantum Institute, University of Maryland, College Park, Maryland 20742, USA; 2 National Institute of Standards and Technology, Gaithersburg, Maryland 20899, USA; 3 Joint Center for Quantum Information and Computer Science, University of Maryland, College Park, Maryland 20742, USA

## Abstract

Correlated phases of matter provide long-term stability for systems as diverse as solids, magnets and potential exotic quantum materials. Mechanical systems, such as buckling transition spring switches, can have engineered, stable configurations whose dependence on a control variable is reminiscent of non-equilibrium phase transitions. In hybrid optomechanical systems, light and matter are strongly coupled, allowing engineering of rapid changes in the force landscape, storing and processing information, and ultimately probing and controlling behaviour at the quantum level. Here we report the observation of first- and second-order buckling transitions between stable mechanical states in an optomechanical system, in which full control of the nature of the transition is obtained by means of the laser power and detuning. The underlying multiwell confining potential we create is highly tunable, with a sub-nanometre distance between potential wells. Our results enable new applications in photonics and information technology, and may enable explorations of quantum phase transitions and macroscopic quantum tunnelling in mechanical systems.

## Abstract (web summary)

Optomechanical systems could form logic gates, but key requirements are two stable static states and the ability to switch between them. Here, the authors observe radiation-pressure induced buckling transitions in an optomechanical system, and control this transition by varying laser power and detuning.

Optomechanical systems provide a unique connection between light and mechanical motion^1^,^2^,^3^ due to both their conceptual simplicity—radiation pressure force induces motion in a compliant optical element—and their practical applications in photonics and sensing^4^,^5^,^6^,^7^,^8^,^9^. A canonical example is the modification of the mechanical spring constant via dynamical effects from the radiation pressure associated with the optical modes coupled to the mechanical system. This so-called ‘optical spring' effect^10^,^11^ has been employed in the contexts of gravity-wave detection^12^, optical trapping of a mirror^13^, raising the mechanical quality factor (‘optical dilution') of a mechanical oscillator^14^ and optical cooling^15^. At the same time, more complex mechanical elements provide new opportunities. For example, nanomechanical devices can be used as memory cells^16^,^17^,^18^ or as logic gates. A crucial ingredient for these applications is to develop a robust element that, when driven electronically or optically, can be set to one of two stable static states. Bistability induced by radiation pressure was demonstrated by Dorsel *et al*.^19^, using a Fabry–Perot cavity in which the length, and thus the resonant frequency, was modified by the circulating optical power. Shortly thereafter, an analogous experiment was performed in the microwave domain^20^. This work was followed by numerous proposals of applications, including the realization of controllable buckled optomechanical systems^21^. Somewhat surprisingly, the only experiments involving optomechanical bistability reported in the meantime have either involved additional electrostatic feedback^22^ or a photothermal mechanism^23^,^24^,^25^ rather than radiation pressure. Instead, dynamical effects, necessary for laser cooling and exploration of narrowband behaviour, have been the focus of researchers in nanoscale optomechanics in recent years. At the same time, static properties beyond bistability provide new application spaces for optomechanics in sensing and optical information processing^21^,^26^. While some experiments with optically driven, pre-buckled devices have yielded successes^18^, the mechanical potential was not optically modified in those systems.

Here we report the observation of radiation pressure-induced buckling transitions in an optomechanical system, an effect predicted several decades ago^21^. Our approach relies on a symmetrical optical cavity with a dielectric membrane in the middle^27^,^28^,^29^, where the behaviour of the mechanical system can be fabricated and characterized separately from the optics necessary to realize an optical cavity of high finesse. Using this platform, we demonstrate an optomechanical system that realizes controlled first- and second-order buckling transitions. These transitions can be understood as arising when the static optomechanical potential changes smoothly from a single-well to a multiwell potential as the optical driving power is increased. Unlike the situation in the pioneering experiment of Dorsel, in which the bistability was associated with a manifestly asymmetric optomechanical potential^21^, our realization results in a spontaneously broken symmetry as the optical drive passes through the transition point. We derive the stability diagram for the buckling transitions, and we show good quantitative agreement between theoretical predictions and experiment.

## Results

### Experimental set-up

**Figure 1:**
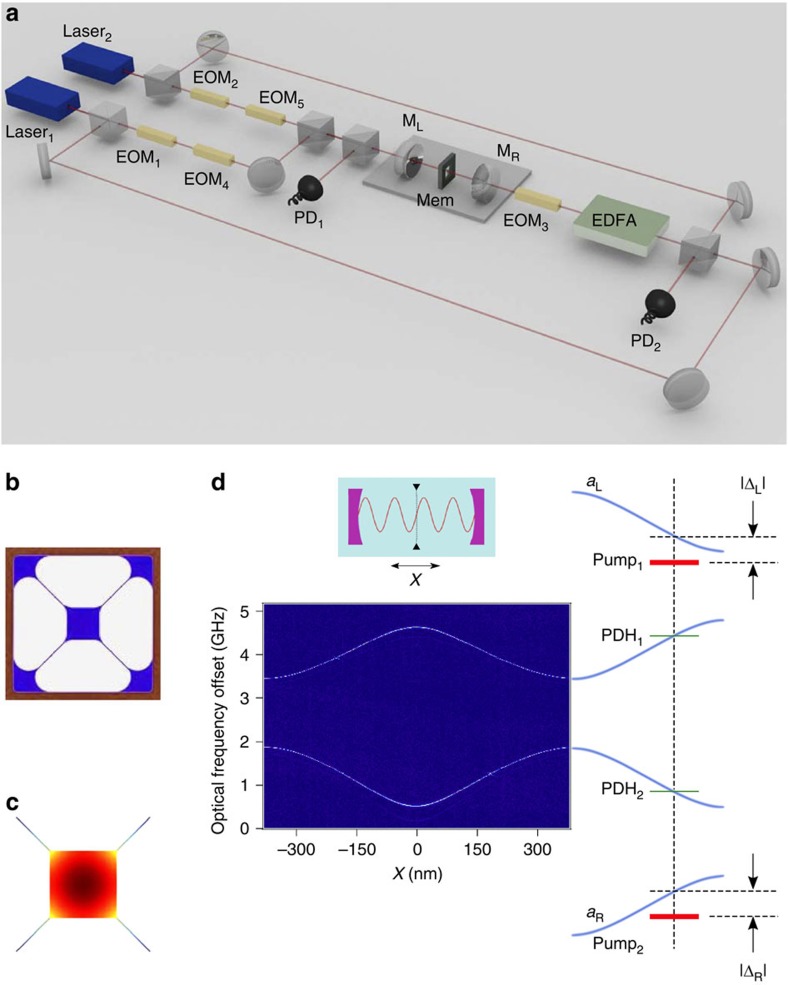
Experimental set-up. (**a**) Curved mirrors M_L_ and M_R_ form a symmetric Fabry–Perot cavity. Tethered membrane Mem that comprises our optomechanical resonator is placed in the centre. The two pump and two probe fields used in the experiment are generated and locked to the cavity by means of independent tunable lasers Laser_1_ and Laser_2_, electro-optic phase modulators EOM_1_–EOM_5_ and an erbium-doped fibre amplifier (EDFA) (details are given in the Supplementary Note 3). Light reflected from the cavity is captured by photodiode PD_1_ to lock the lasers to the cavity, and the beat signal between Laser_1_ and Laser_2_ is captured on PD_2_ to probe the membrane position. (**b**) Microscope image of tethered SiN membrane. The central square is 200 μm on a side. (**c**) Fundamental mechanical mode of membrane determined from finite element analysis; the frequency is 80.3 kHz. (**d**) Optical transmission spectrum for membrane position *x* near the centre of the cavity. Probe lasers PDH_1_ and PDH_2_ are locked to adjacent cavity modes whose resonance frequencies have opposite dependences on membrane displacement. Pump laser Pump_1_ is rigidly offset to probe PDH_1_ and detuned to the red of an adjacent cavity mode by Δ_L_; similarly, pump laser Pump_2_ is detuned to the red of an adjacent cavity mode by Δ_R_, as shown. When the membrane is displaced to the right, Pump_1_ is brought closer to resonance and Pump_2_ is driven further from resonance.

A schematic of our apparatus is shown in Fig. 1a. Two dielectric mirrors, identified as M_L_ and M_R_, form an optical cavity with a length of ∼50 mm. At the centre of the cavity is the optomechanical element, a tensioned silicon nitride membrane (see Supplementary Note 4) normal to the cavity mode with long thin tethers connecting the membrane to its frame. An image taken with an optical microscope is shown in Fig. 1b, and a finite element simulation of the fundamental mechanical mode is shown in Fig. 1c. It has a fundamental mechanical frequency of *ω*_m_=2*π* × 80.3 kHz, designed to allow for substantial optical spring effects at low laser power. The experiment is performed in the classical regime at room temperature and pressure.

Our approach relies on two different optical modes, denoted *a*_L_ and *a*_R_, which have optomechanical couplings of opposite signs. For small displacements of the membrane to the left, mode *a*_L_ is shifted up in frequency while *a*_R_ is shifted down, and vice versa. A two-dimensional experimental plot of cavity transmission versus membrane position and optical frequency is shown in Fig. 1d; the spectrum is periodic with frequency, so for any membrane positions *x* except those corresponding to extrema in the spectrum, there is a large set of pairs of modes with opposite optomechanical couplings *g*_L,R_ (slope of curves in Fig. 1d). The linewidth (FWHM) *κ*/2*π* of the cavity modes is ∼1.8 MHz. We move the membrane about 55 nm away from an anti-crossing, where the optomechanical couplings have amplitudes of *g*_L,R_=±2*π* × 2.1 kHz pm^−1^, and use two such pairs, as illustrated to the right of Fig. 1d. Conceptually, there are four laser fields involved in our experiment, as we now describe.

Two independent probe lasers are locked to a pair of modes with opposite optomechanical couplings by means of the Pound–Drever–Hall (PDH) method, and denoted PDH_1_ and PDH_2_ in Fig. 1d. The probe fields are actually first-order sidebands generated by electro-optic phase modulators EOM_1_ and EOM_2_, as shown in Fig. 1a, on independent lasers denoted ‘Laser_1_' and ‘Laser_2_'. (A detailed description of how the laser fields are generated can be found in the Supplementary Note 3 and Supplementary Fig. 1). Due to the opposite signs of the optomechanical couplings of the modes to which the probe fields are locked, the frequency difference between PDH_1_ and PDH_2_ is proportional to the membrane displacement, with twice the response of either mode alone. We access this frequency difference by counting the beat signal between the two lasers as detected with photodetector PD_2_. At the 2 kHz data acquisition rate employed in this work, the position measurement resolution is better than 1 pm.

We supplement the probe fields with additional fields to create a tailorable, multiwell optomechanical potential. The low coefficient for the absorption of light in silicon nitride at our wavelength (*λ*=1,550 nm), combined with the active cooling provided by the ambient air environment, leaves us dominated by radiation pressure for the effective potential (see Supplementary Note 6 and Supplementary Fig. 2). Two strong pump fields, denoted Pump_1_ and Pump_2_ in Fig. 1d, are generated by combining light from the lasers, amplifying it, and passing it through phase modulator EOM_3_. Pump_1_ has power *P*_L_ and (angular) frequency *ν*_L_ and is frequency-offset from probe field PDH_1_ by ∼2 GHz, the sum of the drive frequencies for EOM_1_ and EOM_3_, such that it drives mode *a*_L_ with (at low pump power) red detuning Δ_L_. Pump_2_ has power *P*_R_ and angular frequency *ν*_R_ and is similarly offset from probe PDH_2_ so as to drive mode *a*_R_ with detuning Δ_R_. Crucially, the optomechanical coupling of each pump field is of the opposite sign of the probe field to which it is frequency-offset. While a rich variety of phenomena is accessible by taking independent values of *P*_L_, Δ_L_, *P*_R_ and Δ_R_, the experiments described here employ the symmetric situation *P*_L_=*P*_R_ and Δ_L_=Δ_R_.

### Theoretical analysis

In the symmetric configuration, there is only one stable steady state for low to intermediate pump power levels. At higher powers, however, additional steady states appear. For the symmetric case we have constructed, the solutions to the dynamical equations describing these steady states have a particularly simple form. The optical fields *a*_L_ (left-moving) and *a*_R_ (right-moving) take the form of coherent states, with amplitudes


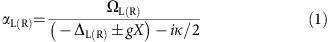


where Ω_L(R)_ are related to the incident laser powers by Ω_L(R)_=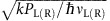, and *X* is the steady state displacement of the membrane, including the fundamental and higher modes. We note that the fact that the PDH lock tracks the changes in the cavity frequencies leads to a shift *ν*→*ν*±*gX* for the steady state, effectively enhancing the low-frequency contribution to *g* by a factor of 2.

As in the experiment, we only focus on the lowest frequency mechanical mode and symmetric driving and detuning, Ω_L(R)_=Ω, Δ_L(R)_=Δ. This mode feels a radiation pressure force and a spring-based restoring force with spring constant *k*=*m*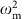, and the steady state is determined by points where the total force is zero and restorative under small variations in *X*. Qualitatively, the membrane's motion evolves in a potential combining its internal spring and two competing optical springs. Zeros in the force (minima and maxima of the potential) occur when


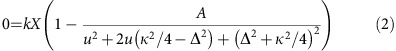


where *u*=(2*gX*)^2^ is the square of the frequency shift including PDH feedback, and the parameter 
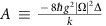
 is proportional to the pump power.

**Figure 2:**
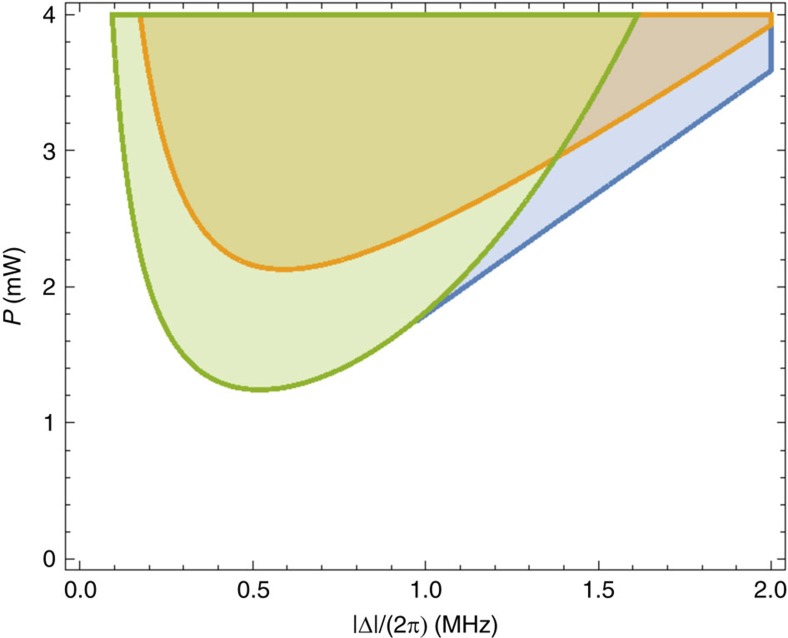
Theoretical stability diagram. Second-order (green) and first-order (blue) buckling transitions as a function of laser detuning and power *P*=*P*_L_=*P*_R_ are shown. In addition to the stable solutions, the orange overlay indicates the region where radiation pressure provides mechanical gain, raising the quality factor of the mechanical oscillator and potentially causing mechanical instability. Experimental data were taken at detunings Δ/2*π*=−0.4 and −1.2 MHz.

This equation has solutions for *u*=0 and for 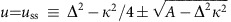; physical solutions require *u*≥0. Implicitly this requires *A*>Δ^2^*κ*^2^, so that *u*_ss_ is real. For Δ<*κ*/2, there is only one non-zero *u* solution, and the system continuously goes, as a function of power, from *X*=0 to 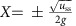. This is in direct analogy to a second-order buckling transition in a spring, where the broken left/right symmetry is evident even for arbitrarily small values of the displacement. For Δ>*κ*/2, however, there are two solutions for *u*_ss_. The smaller corresponds to an unstable branch, while the larger is stable. This leads to a discontinuous change of the membrane displacement at the transition radiation pressure, and provides a first-order buckling transition in the optomechanical system. The overall stability diagram (see Supplementary Note 1 for derivation), including the experimentally probed regimes, is shown in Fig. 2. Both first- and second-order transitions occur, depending upon the detuning, for increasing power. At sufficiently large power levels, the dynamical effects associated with the radiation pressure provide mechanical gain, leading to instability if it overcomes the atmospheric damping. The focus of this work is to study the system at power levels below this threshold.

### Experimental results

**Figure 3:**
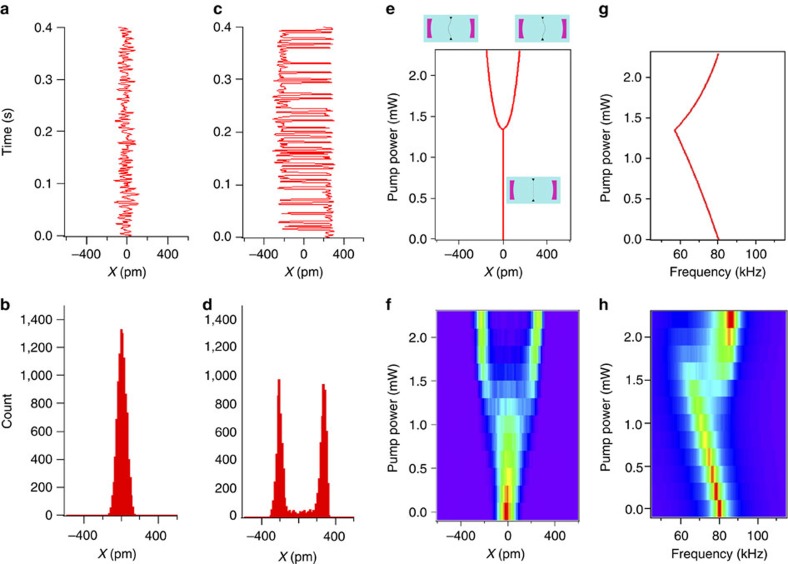
Second-order buckling transition. (**a**) Real-time data of the membrane position without pump lasers. (**b**) Corresponding histogram. The membrane fluctuates around a single position, resulting from a single-well mechanical potential. (**c**) Real-time data of the membrane position with detuning Δ=0.22*κ* and pump power *P*_L_=*P*_R_=2.2 mW. (**d**) Histogram of the membrane position with detuning Δ=0.22*κ* and *P*_L_=*P*_R_=2.2 mW. The membrane fluctuates around two stable positions, resulting from a double-well optomechanical potential; the fluctuations are largely induced by mechanical noise in the positioning stage supporting the membrane. (**e**) Calculated stable positions as a function of pump laser power. (**f**) Image of experimental histograms of the membrane position for increasing pump power. The single-well potential develops smoothly into a double-well potential as the power is raised, showing the onset of the second-order buckling transition. (**g**) Calculated mechanical frequency of the membrane for small excursions about the stable positions as a function of pump power. (**h**) Image of mechanical power spectral density inferred from experimental data. The frequency drops as the global potential well initially becomes more shallow, then increases as the membrane buckles into a local potential minimum.

We now show the experimental buckling of the optically sprung membrane for Δ=2*π* × 0.4 MHz=0.22*κ*, where we expect that the dynamics will correspond to a second-order buckling transition. In the absence of a pump laser, the membrane experiences a pure mechanical single-well quadratic potential and fluctuates around the stable position due to both thermal and technical noise. We record the position of the membrane at a sampling rate of 2 kHz for 5 s (see Supplementary Note 5 for position readout); Fig. 3a shows a characteristic subset of the data for 0.4 s, and Fig. 3b shows a histogram of the complete data set, peaked around zero displacement as expected. When the pump fields are turned on, with pump power *P*_*L*_=*P*_*R*_=2.2 mW, the membrane fluctuates around two stable positions, as shown in the time series in Fig. 3c. We attribute the jumping between stable positions primarily to mechanical noise in the nanopositioning stage holding the tethered membrane. As shown in the histogram of the membrane position in Fig. 3d, the membrane buckles to either left or right. As the pump power is raised, the dwell times in the buckled states increase. Figure 3e shows the steady state positions predicted by theory, and implicit in the stability diagram shown in Fig. 2, as a function of pump laser power. Corresponding experimental histograms of the membrane position are shown for the same range of pump powers in Fig. 3f. Both theory and experiment indicate an apparent second-order transition in the membrane displacement *X* as the pump power is raised.

Accompanying the spontaneously broken spatial symmetry as the system passes through the buckling transition is a change in the system dynamics. We observe this by analysing the spectrum of the PDH signal for frequencies higher than the bandwidth (∼3 kHz) of the servos used to lock the lasers to the cavity. Figure 3h shows that as the pump power is raised, the frequency of the optically sprung resonator initially diminishes, and then rises above the frequency of the bare mechanical resonator as the system buckles. This is consistent with the picture that the potential experienced by the membrane evolves from a single well to a double well, the sum of the mechanical and optical potentials.

Curiously, the frequency of the mechanical mode does not go all the way to zero at the buckling transition, as might be expected. This is a consequence of the limited bandwidth of the feedback electronics used to lock the probe lasers to the optical resonances. Specifically, the opposite frequency dependence with position of the pump lasers and their associated probes (Fig. 1d) results in a doubling of the optomechanical coupling for displacements within the bandwidth (∼3 kHz) of the feedback electronics, relative to displacements at substantially higher frequencies. Since the mechanical frequency of the membrane is far above this cutoff, the optical power required to buckle the membrane is well below the power required to drive its frequency to zero in the unbuckled state. Qualitative agreement with the single mechanical mode theory (including the effect of the feedback; see Supplementary Note 2) is obtained and shown in Fig. 3g, but quantitative agreement likely will require inclusion of the higher mechanical modes whose properties remain challenging to fully characterize in the present set-up.

**Figure 4:**
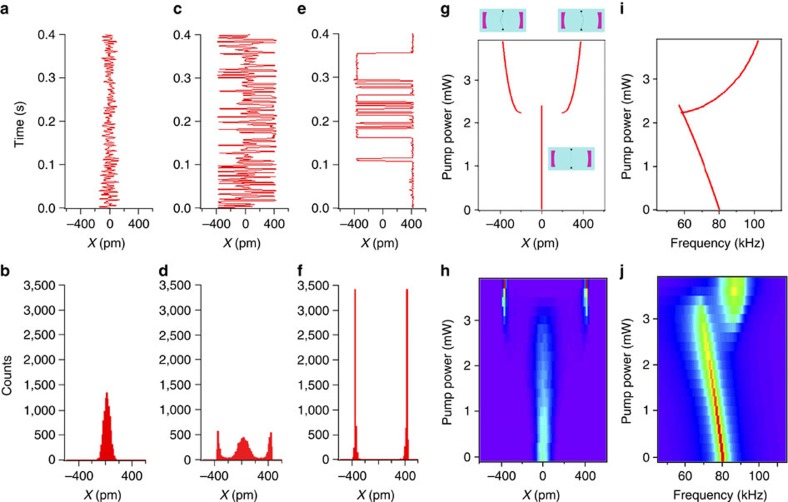
First-order buckling transition. (**a**) Time series and (**b**) histogram of membrane position, no pump. (**c**) Real-time data and (**d**) histogram of the membrane position with detuning Δ=0.67*κ* and pump power *P*_L_=*P*_R_=3.0 mW. The membrane fluctuates around three stable positions, resulting from a triple-well optomechanical potential. (**e**) Real-time data and (**f**) histogram of the membrane position with detuning Δ=0.67*κ* and *P*_L_=*P*_R_=3.8 mW. The membrane now fluctuates around just two stable positions, due to a double-well optomechanical potential. (**g**) Calculated stable positions as a function of pump laser power. For a small range of pump powers, there are three stable positions, and as the pump power is raised, the unbuckled state becomes unstable. (**h**) Image of experimental histograms of the membrane position for increasing pump power. (**i**) Calculated mechanical frequency of the membrane for small excursions about the stable positions as a function of pump power. (**j**) Image of mechanical power spectral density inferred from experimental data.

Based on the theoretical model underlying the stability diagram of Fig. 2, we expect the buckling transition to be qualitatively different for detunings Δ≥*κ*/2. Figure 4 shows our examination of the buckling transition for Δ=2*π* × 1.2 MHz=0.67*κ*. Once again, Fig. 4a,b depicts the noise-induced fluctuations of the membrane in the absence of pump lasers. Figure 4c,d shows the time series and histograms of membrane position for *P*_L_=*P*_R_=3.0 mW; this time, the membrane fluctuates around three stable positions, either remaining unbuckled or buckling to either left or right. When the pump powers are raised to 3.8 mW, only the buckled states remain stable, as shown in Fig. 4e,f.

Theoretical predictions of the steady states as a function of pump power are shown for our experimental conditions in Fig. 4g, and the corresponding experimental histograms of the membrane position are shown in Fig. 4h. In addition to the initial unbuckled state, two more stable positions appear discontinuously as the pump power is increased, indicating that the membrane now experiences an effective triple-well potential. This jump to a finite displacement of the membrane indicates a non-equilibrium first-order buckling transition. As the power is raised still further, the steady state at zero displacement becomes unstable, and the potential becomes a double well. We note that the strong quantitative agreement of our radiation pressure model with these results confirms our neglect of photothermal terms.

Examination of the mechanical oscillation frequency, Fig. 4j, once again reveals that the frequency of the optically sprung resonator initially diminishes with pump power, but jumps to a value larger than the bare mechanical frequency in the final, double-well regime. For a small range of powers, corresponding to the triple-well regime, the frequency distribution is bimodal. The corresponding theoretical curve, including feedback as discussed previously, is shown in Fig. 4i, where it is clear that different mechanical frequencies are expected in the local potential minima corresponding to the buckled and unbuckled states in the triple-well potential regime. We note that the power levels chosen push into the nominally unstable region of the stability diagram shown in Fig. 2 for the large detuning data. Associated limit cycle behaviour leads to deviations in the experiment from the simple theoretical picture presented earlier, as the system wanders in a potential landscape with position-dependent gain and loss. We believe that this fact, coupled with the feedback and the restriction of our theoretical model to a single mechanical mode, are responsible for the differences in the shapes of the experimental data in Fig. 4h,j from their theoretical counterparts Fig. 4g,i.

## Discussion

Our results, which can be generalized to other optomechanical systems, suggest a variety of applications. For laser power near the buckling transition, the response of the membrane to external forces increases dramatically, enabling higher sensitivity for force or acceleration sensing. At higher laser powers, the membrane dwells in its local potential well for significant times, enabling operation as an optomechanical memory, in which the position of the membrane can be switched between the wells by a single laser pulse. Jumps between the potential wells seen in this work arise from technical noise, and may be suppressed by increasing the laser power, using lower-noise positioning stages, and working at low temperature. Our system also provides a platform for studying nonlinear optomechanics and chaotic dynamics, such as dynamical multistability^30^. Furthermore, at low temperatures and with a high mechanical quality factor, a quantum phase transition may be observable in systems of this nature^31^. Specifically, an optomechanical system can be made sufficiently cold—with a nominal dephasing rate slower than its resonance frequency—and sideband resolved to be laser cooled to its ground state before buckling^32^. Then a rapid increase in pump power bringing the system across the stability boundary could yield a transition driven entirely by quantum fluctuations, a macroscopic version of structural quantum phase transitions such as those in ion crystals^33^.

### Data availability

Data are stored and saved according to US government policy, and can be made available by request to the authors on an individual basis.

## Additional information

**How to cite this article:** Xu, H. *et al*. Observation of optomechanical buckling transitions. *Nat. Commun.* **8,** 14481 doi: 10.1038/ncomms14481 (2017).

**Publisher's note:** Springer Nature remains neutral with regard to jurisdictional claims in published maps and institutional affiliations.

## Supplementary Material

Supplementary Information Supplementary Figures and Supplementary Notes
