# Supplementary material for: Observation of optomechanical buckling transitions
Source: Nat Commun. 2017 Mar 1;8:14481. doi: 10.1038/ncomms14481 (PMC5337942; doi:10.1038/ncomms14481)
Supplement: Supplementary Information — Supplementary Figures and Supplementary Notes [file ncomms14481-s1.pdf]

Supplementary Note 1. Derivation of the stability diagram

Consider a two-mode optical system where the optical frequency difference is much larger than any mechanical scale in the problem. We can then neglect beating between the two optical fields, and work with each cavity mode rotating in a frame near resonant with the closer of the two pump lasers with Rabi frequencies $\Omega_{\rm L}$, $\Omega_{\rm R}$. Labeling the modes L and R (where L represents a mode that increases in frequency as the mechanical oscillator moves leftward, and R the opposite), we have equations of motion for the fields $a_{\rm L}, a_{\rm R}$ in this rotating frame:

$$\dot{a}_{\rm L} = i(\Delta_{\rm L} - g_{\rm L}x)a_{\rm L} - \frac{\kappa_{\rm L}}{2}a_{\rm L} + \sqrt{\kappa_{\rm L}}a_{\rm L,in} + i\Omega_{\rm L} \tag{1}$$

$$\dot{a}_{\rm R} = i(\Delta_{\rm R} + g_{\rm R}x)a_{\rm R} - \frac{\kappa_{\rm R}}{2}a_{\rm R} + \sqrt{\kappa_{\rm R}}a_{\rm R,in} + i\Omega_{\rm R} \tag{2}$$

where $\Delta_{\rm L} = \nu_{\rm L} - \omega_{\rm L}$ is the detuning between the L pump laser and the cavity L mode (initially negative for our setup), $a_{\rm L(R),in}$ are the input vacuum fields neglected in the classical analysis that follows, and x is the operator representing the displacement of the mechanical oscillator along the cavity axis relative to the zero pump power position, which in general involves the motion of multiple mechanical modes. The coupling constants $g_{\rm L}, g_{\rm R}$ are measured in the experiment by observing the change of angular frequency for the cavity as a function of position of the membrane. We neglect terms beating at the laser frequency difference ($\sim$ 8 GHz, and thus faster than any other scale in the problem). In the experimental setup, $\kappa_{\rm L} \sim \kappa_{\rm R} = \kappa$. The radiation pressure force is $F_{\rm rad} \approx -\hbar(g_{\rm L}a_{\rm L}^\dagger a_{\rm L} - g_{\rm R}a_{\rm R}^\dagger a_{\rm R})$. Note that for our system $g_{\rm L} = g_{\rm R} = g$, and we similarly set $\kappa_{\rm L} = \kappa_{\rm R} = \kappa$ for simplicity, neglecting potential dispersive effects.

When an optical system is pumped with a laser system, the behavior is best described by finding the classical steady state of the system and looking at fluctuations around that steady state. For simplicity, we start with the scenario where only the fundamental mechanical mode with spring constant k is coupled to the optical system. We can look for the steady state of the driven system by solving the equations for coherent state amplitudes $\alpha_{\rm L}, \alpha_{\rm R}$. This yields

$$\alpha_{\rm L(R)} = \frac{\Omega_{\rm L(R)}}{(-\Delta_{\rm L(R)} \pm gX) - i\kappa/2} \tag{3}$$

where X is the steady-state position of the mechanical resonator.

To go from the cavity amplitudes to the full steady state requires examining both the cavity and the mechanical equations of motion. In practice, our description must also include the action of the feedback electronics, particularly if we want to know about the dynamics of our system. Fortunately, inclusion of the feedback can be included with a simple model with an additional degree of freedom δ representing the action of laser locking by the probe beams, which leads to slow feedback in the detunings of the pump fields. The equations become

$$\dot{x} = p/m \tag{4}$$

$$\dot{p} = -kx - \gamma_{\rm m}p + F_{\rm rad} \tag{5}$$

$$\dot{\delta} = -\frac{1}{\tau_{\rm FB}}(\delta - gx) \tag{6}$$

$$\Delta_{\rm L} = \Delta - \delta \tag{7}$$

$$\Delta_{\rm R} = \Delta + \delta \tag{8}$$

where $\tau_{\rm FB} \gg \sqrt{k/m}$ is the feedback timescale, around 100 µs in the experiment, and Δ is the bare detuning offset set by the EOMs.

On very slow time scales, the system goes to a steady state with $\delta_{\rm ss} = gX$ (enabling readout of the position of the resonator using the feedback circuit). In practice, the effect of the feedback will be to double the optomechanical response in the system. The oscillator position is found by solving for the zero force condition

$$kX = -\hbar g(|\alpha_{\rm L}|^2 - |\alpha_{\rm R}|^2) \tag{9}$$

For the symmetric case, finding the steady state corresponds to solving a surprising simple equation for zero force. The zero force condition becomes

$$0 = kX\left(1 + \frac{8\hbar g^2\Delta\Omega^2}{k}\frac{1}{\kappa^4/16 + (4g^2X^2 + \Delta^2)\kappa^2/2 + (\Delta^2 - 4g^2X^2)^2}\right) \tag{10}$$

This has the trivial (low power) solution $X = 0$. In addition, at higher power it admits additional solutions.

To examine the higher power solutions, we define $u \equiv (2gX)^2$ as a generalized position variable and $A \equiv -8\hbar g^2|\Omega|^2\Delta/k$ as a variable proportional to the incoming optical power. Note that $\Delta < 0$ (red detuning) for the experiment, and thus $A > 0$. The zero force condition has solutions in u according to the quadratic equation:

$$u_{\mathrm{ss},\pm} = \Delta^2 - \kappa^2/4 \pm \sqrt{A - \Delta^2\kappa^2} \tag{11}$$

Solutions to these equations provide the stability diagram shown in the main text.

We examine which of these solutions is physical. We require X to be real, or equivalently $u > 0$ and real. If $|\Delta| < \kappa/2$, only the positive solution $u_{\mathrm{ss},+}$, can satisfy $u > 0$. At the critical value of $A = A_2 \equiv (\Delta^2 + \kappa^2/4)^2$, $u_{\mathrm{ss},+} = 0$, and above this, u continuously takes a non-zero value. This corresponds to the second-order buckling transition as described in the main text, and can be understood as a double well potential for X

When $|\Delta| > \kappa/2$, both branches may satisfy $u > 0$. The requirement of u real is equivalent to $A > A_1 \equiv \Delta^2\kappa^2$, at which u discontinuously takes a non-zero value. This corresponds to the first-order buckling transition as described in the main text. This second regime corresponds to a triple well potential for X, with the smaller solutions for $u \neq 0$ unstable (the peaks of the barriers between wells). Thus, if we conceive of a non equilibrium stability diagram in which optical power is varied, for small detuning a second order buckling transition will occur, while for larger detunings, a first order (discontinuous change of X as a function of A) transition occurs.

One potential limit to stability for these systems is the detuning of the cavity modes becoming sufficiently modified by the transition to go from 'red' to 'blue'. This occurs when $2g|X| > |\Delta|$ or $u > \Delta^2$, which occurs for

$$A > A_{\mathrm{inst}} \equiv \kappa^4/16 + \Delta^2\kappa^2 \ . \tag{12}$$

In practice, the stability of the overall system can be maintained even when one of the cavity modes is blue detuned, given sufficient mechanical damping. In addition, we find via numerical simulations that larger values of A lead to limit cycle behavior that behaves similarly to the steady state solutions already found.

Supplementary Note 2. Dynamical response

In addition to the steady state solutions, we can examine the dynamical response of the system near its steady state. As our system is operating in the limit of cavity linewidth much greater than mechanical frequency, we anticipate that the dominant corrections to the bare resonator behavior take the form of the optical spring effect.

Formally, we can find this behavior via expansion of the equations of motion for small excursions from the steady state solutions. Specifically, for equations of the motion of the form $\dot{v}_\mu = M_\mu(\vec{\mathbf{v}}) - F_\mu$, such as those given in Eqs. 1-2,4-6, we find the steady state $\vec{\mathbf{v}}$ and expand around it with $\delta\vec{\mathbf{v}}$. The corresponding equations of motion are

$$\dot{\delta v}_\mu = (\partial_\sigma M_\mu|_{\vec{\mathbf{v}}})\delta v_\sigma \tag{13}$$

where the Einstein summation condition is implied for Greek indices μ, σ. Moving to the Fourier domain with frequency coordinate ν, we can eliminate the equations involving fluctuations of the cavity fields, finding they directly depend upon the position fluctuations x and the detuning fluctuations δ.

Taking $\tau_{\mathrm{FB}} \gg 1/\nu$ but $\nu \ll \kappa$, we can expand the corrections from the cavity coordinates to recover the optical spring result: the spring constant k is modified, as is the mechanical damping γ and the effective inertial mass m:

$$k_{\text{eff}} = k + 2g^2\hbar\left(\frac{|\alpha_{\text{L}}|^2(\Delta - 2gX)}{(\Delta - 2gX)^2 + \kappa^2/4} + \frac{|\alpha_{\text{R}}|^2(\Delta + 2gX)}{(\Delta + 2gX)^2 + \kappa^2/4}\right) \tag{14}$$

$$\gamma_{\text{eff}} = \gamma + \frac{2}{m}\partial_\kappa k_{\text{eff}} \tag{15}$$

$$m_{\text{eff}} = m + 2\partial_\kappa^2 k_{\text{eff}} \tag{16}$$

where partial derivatives assume that steady state values $\alpha_{\text{L}}, \alpha_{\text{R}}, X$ are independent of κ. In practice, for our parameters, the difference in the effective mass is negligible. These formula are then used to find the dynamical response and the stable region of the stability diagram given in the main text. In contrast to the case without feedback, here feedback causes the mechanical mode to have a non-zero frequency at the buckling transition.

Supplementary Note 3. Generation of pump and probe fields

We clarify here how the pump and probe fields represented in Fig. 1d of the main text are generated. Laser_1 and Laser_2 are independent tunable lasers with $\lambda = 1560$ nm. Referring to Supplementary Figure 1, electro-optic phase modulators EOM_1 and EOM_2 are driven independently with frequencies in the vicinity of 1.5 GHz, and generate sidebands on Laser_1 and Laser_2. The lower-frequency sideband of EOM_1 is denoted PDH_1 and locked to one mode of the cavity, and the upper-frequency sideband of EOM_2 is denoted PDH_2 and locked to an adjacent mode. Locking is accomplished by the Pound-Drever-Hall (PDH) method, using electro-optic modulators EOM_4 and EOM_5 to generate sidebands (not shown in the figure) at 20 MHz. Part of the light from Laser_1 and Laser_2 is combined and amplified in an erbium-doped fiber amplifier and passed through phase modulator EOM_3, driven at a frequency of approximately 0.5 GHz. The upper-frequency sideband of Laser_1 is denoted Pump_1 and the lower-frequency sideband of Laser_2 is denoted Pump_2. The detunings Δ_{L} and Δ_{R} (taken to be equal in the experiment) of the pump fields from their associated cavity modes may be controlled independently by choice of the frequencies driving EOM_1 and EOM_2. The probe fields are combined and sent into one port of the cavity, and the pump beams are combined and sent into the other port.

Supplementary Note 4. Fabrication of tethered membrane

We start with a double side polished silicon wafer as the substrate. First we deposit a layer of silicon nitride film on both sides of the wafer using a low pressure chemical vapor deposition furnace. The thickness of the silicon nitride film is measured to be about 258 nm by a spectroscopic ellipsometer, limited by the instrumental resolution. Then we do photolithography and plasma etching to open a square window in the silicon nitride film on one side of the wafer. Removing the silicon in the window by wet etching using potassium hydroxide solution, we obtain a square suspended silicon nitride membrane. The membrane is furthered shaped to a tethered membrane (200 µm × 200 µm) with long thin tethers (2 µm wide) connecting the membrane to its frame using electron-beam lithography and plasma etching. This is done to reduce the effective spring constant and allow for a substantial optical spring effect at low laser power. Finite element analysis is used to simulate the mechanical properties of the tethered membrane to aid in the design.

Supplementary Note 5. Position readout of the tethered membrane

We use two different schemes for the measurement of small membrane position variations. For measurements in a bandwidth below 1 kHz, we employ the fact that the variation of the beat frequency between the probe lasers is locally proportional to the variation of the membrane position. Since the probe fields contain additional frequency components generated by EOMs, we instead measure the beat frequency between the (locked) original lasers,

whose frequencies directly track those of the probes. The beat frequency measurement is performed with a high-frequency counter. We use this approach for low-frequency measurements, such as in measuring the stable position of the membrane before and after the buckling transitions.

For measuring mechanical motion at frequencies substantially higher than the PDH servo bandwidth (~ 3 kHz), we use the PDH signal as a probe of membrane position variation. The power spectral densities shown in Fig. 3h and Fig. 4j are obtained by Fourier transform of the PDH signal.

Supplementary Note 6. Photothermal considerations

We have used finite-element simulations (COMSOL Multiphysics®) to model the effect of material absorption on the membrane temperature and the implications this has for our experiments.

An upper bound on the optical power within the cavity is obtained by considering the resonant optical power circulating in the cavity without the membrane, in conjunction with the absorption of silicon nitride. The finesse of the empty cavity is approximately $F = 2\,000$, and the largest input power we quote is $P_{\text{in}} = 3.8$ mW. Assuming perfect mode-matching, an upper bound to the circulating power is then $P_{\text{circ,max}} = (F/\pi)P_{\text{in}} = 2.4$ W. Taking a conservative value for the imaginary part of the index of refraction of silicon nitride at 1550 nm to be $n_{\text{I}} = 1.6 \times 10^{-5}$, we find an upper bound for the absorbed power to be 0.2 mW.

We model a square membrane, 200 µm on a side and 258 nm in thickness, attached at the corners to a thermal bath at temperature $T_{\text{amb}} = 293.15$ K by four tethers of length 260 µm and cross-sectional area 2 µm $\times$ 0.258 µm. The entire system is enclosed in a spherical volume of air of radius 800 µm, with the temperature fixed at T_{amb} at the boundary. We consider only thermal conduction, neglecting radiation and convection; the membrane heating that we find is thus a conservative upper bound. The thermal conductivity of the air is taken to be $0.0257\,\text{W}\,\text{m}^{-1}\text{K}^{-1}$, while values of the thermal conductivity of silicon nitride have been reported spanning the range of $10\,\text{W}\,\text{m}^{-1}\text{K}^{-1} - 50\,\text{W}\,\text{m}^{-1}\text{K}^{-1}$. We apply heat sources with a Gaussian spatial distribution of standard deviation $\sigma = 12.5$ µm and power 0.1 mW centered on each side of the membrane, corresponding to an optical waist of $\omega_0 = 25$ µm. Taking the thermal conductivity of silicon nitride to be $10\,\text{W}\,\text{m}^{-1}\text{K}^{-1}$, we find the temperature distribution shown in Supplementary Figure 2a. The spatially averaged rise in the membrane temperature is $\Delta T_{\text{avg}} = 8.38$ K, ranging from $\Delta T_{\text{center}} = 21.4$ K at the center to $\Delta T_{\text{corner}} = 4.74$ K at the corners. The corresponding values for calculations done with a silicon nitride thermal conductivity of $50\,\text{W}\,\text{m}^{-1}\text{K}^{-1}$ are $\Delta T_{\text{avg}} = 7.46$ K, $\Delta T_{\text{center}} = 10.94$ K, and $\Delta T_{\text{corner}} = 5.94$ K.

The mechanical restoring force in our tethered membrane is provided by the tension in the silicon nitride tethers, arising from the difference in the thermal expansion coefficients of silicon nitride and the silicon wafer on which it is deposited as the system is cooled from $T_{\text{fab}} \approx 800$ °C to room temperature. The fractional change in the tension when the tethers are warmed to temperature $T(x)$ from ambient temperature T_{amb} is

$$\frac{\delta F}{F} = -\frac{\bar{T} - T_{\text{amb}}}{T_{\text{fab}} - T_{\text{amb}}}$$

where $\bar{T}$ is the average of $T(x)$ over the length of a tether. The corresponding fractional change in mechanical frequency ν_m is

$$\frac{\delta \nu_m}{\nu_m} = \frac{1}{2}\frac{\delta F}{F}$$

Supplementary Figure 2b shows the calculated radial temperature distribution, including the tethers, for assumed silicon nitride thermal conductivities of $10\,\text{W}\,\text{m}^{-1}\text{K}^{-1}$ and $50\,\text{W}\,\text{m}^{-1}\text{K}^{-1}$. Taking the thermal conductivity to be $10\,\text{W}\,\text{m}^{-1}\text{K}^{-1}$, our simulations show the spatially averaged temperature rise in the tethers $\bar{T} - T_{\text{amb}}$ to be 1.48 K, from which we infer a fractional reduction in the mechanical frequency of at most $\delta\nu_m/\nu_m = -9.4 \times 10^{-4}$.

The corresponding result for a silicon nitride thermal conductivity of $50\,\mathrm{W\,m^{-1}K^{-1}}$ is $\delta\nu_m/\nu_m = -1.1\times10^{-3}$. In either case, the frequency shift is negligible compared to the one induced by radiation pressure.

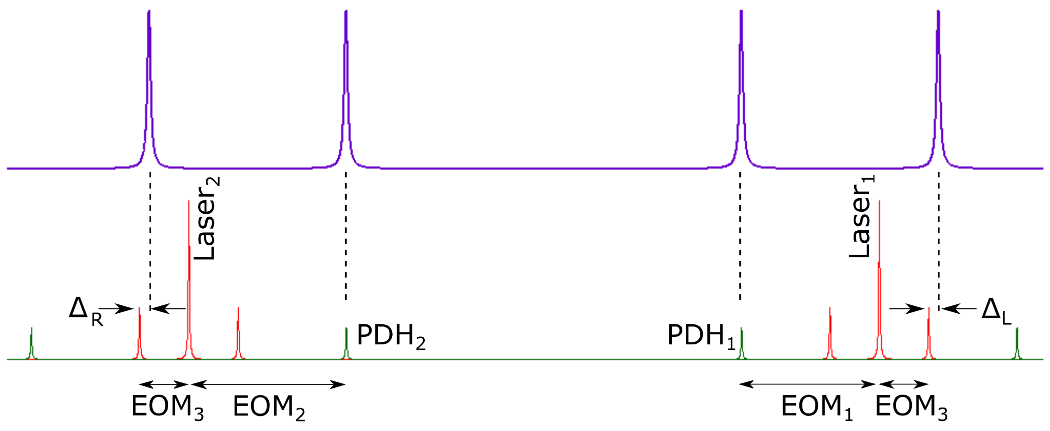

Supplementary Figure 1: **Generation of pump and probe laser fields.** The relevant cavity modes (top), and spectrum showing how pump and probe laser fields are generated with electro-optic phase modulators (bottom). Probe fields PDH_1 and PDH_2 are first-order sidebands (shown in green) on lasers Laser_1 and Laser_2, generated by electro-optic modulators EOM_1 and EOM_2, respectively. One pump field, red-detuned from a cavity resonance by Δ_L, is a first-order sideband on Laser_1 generated by electro-optic modulator EOM_3. The other pump field, red-detuned from a cavity resonance by Δ_R, is a first-order sideband on Laser_2 also generated by EOM_3. Laser fields that are not near-resonant with cavity modes are rejected by the cavity and play no role. In the interest of clarity, sidebands generated for the purpose of obtaining a Pound-Drever-Hall error signal, generated by electro-optic modulators denoted by EOM_4 and EOM_5 in Figure 1 of the main text, are not shown.

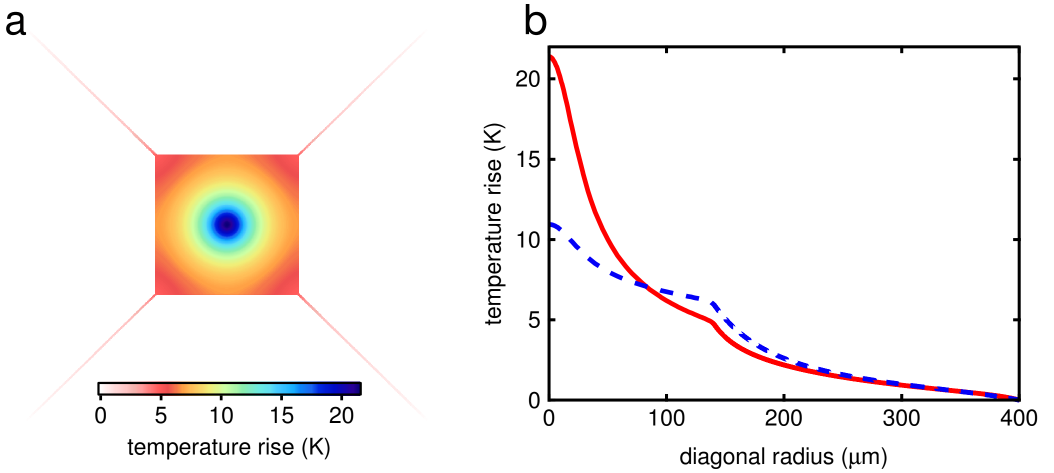

Supplementary Figure 2: **Finite-element calculation of absorptive heating.** **(a)** Rise in temperature (K) arising from absorption, taking a pump power of $P_\mathrm{in} = 3.8$ mW and a silicon nitride thermal conductivity of $10\,\mathrm{W\,m^{-1}K^{-1}}$. **(b)** Radial temperature distribution calculated along the diagonals, including the tethers ($r > 141$ μm). The red solid curve shows the temperature distribution assuming a thermal conductivity of $10\,\mathrm{W\,m^{-1}K^{-1}}$, and the blue dashed curve shows the temperature distribution assuming a thermal conductivity of $50\,\mathrm{W\,m^{-1}K^{-1}}$.
